# Rivaroxaban triggered multifocal intratumoral hemorrhage of the cabozantinib-treated diffuse brain metastases: A case report and review of literature

**DOI:** 10.1515/med-2021-0261

**Published:** 2021-04-09

**Authors:** Luyue Chen, E Chen, Yanlin Huang, Xinhua Tian

**Affiliations:** Department of Neurosurgery, Zhongshan Hospital Xiamen University, Xiamen, 361004, Fujian, People’s Republic of China

**Keywords:** brain metastases, rivaroxaban, intratumoral hemorrhage, venous thromboembolism

## Abstract

Brain metastases (BMs) are the most common intracranial malignancy with poor prognosis. Patients with intracranial tumors are at greater risk for thrombotic complications and intracranial hemorrhage. Rivaroxaban is a potent oral anticoagulant with the high selectivity of direct factor Xa inhibition. The incidence and severity of rivaroxaban-triggered intratumoral hemorrhage (ITH) in patients with BMs remain unknown. A 57-year-old woman was diagnosed with multiple lung, bone, and BMs from unknown primary cancer origin, and refused any invasive procedures to confirm tumor pathology. However, this patient had a relatively favorable outcome after treating with cabozantinib, an inhibitor of multiple tyrosine kinases. The patient survived over 2 years and developed deep vein thrombosis of right lower limb. Oral rivaroxaban was prescribed, and the multifocal catastrophic ITH was encountered after 1 week. The last head computed tomography imaging revealed a rare but typical image of diffuse hemorrhagic metastases. Hemorrhagic-prone BMs, therapeutic rivaroxaban, and cabozantinib treatment increase risks to develop ITH. In this case rivaroxaban was the trigger to this terminal event. This case is a miserable lesson and keeps reminding us to stay vigilant in clinical practice even when there is a potential benefit for anticoagulation in such population.

## 1 Introduction

**Figure 1:**
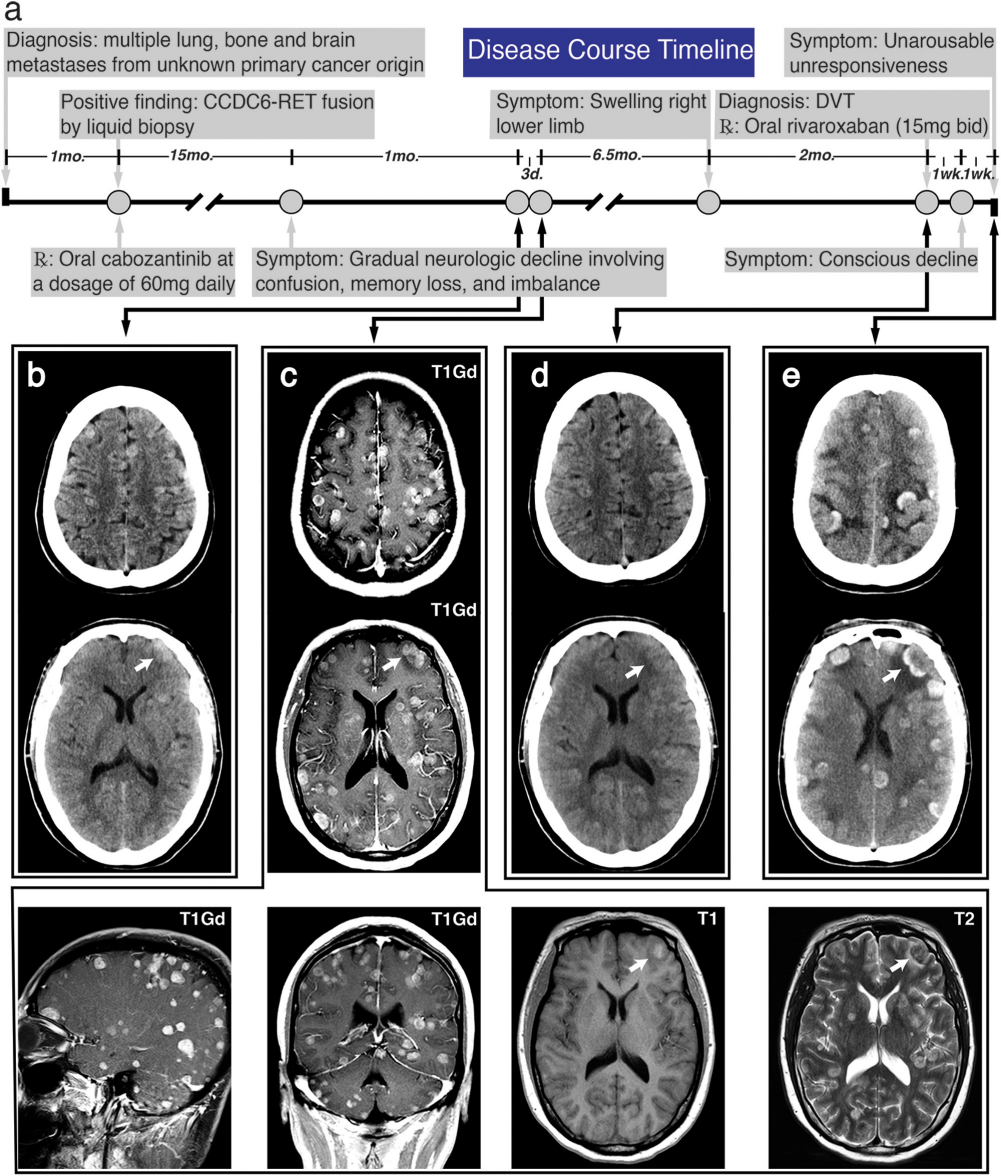
Timeline of major disease-related events and representative images of head imaging. (a) A schematic timeline displayed the major events during disease progression. (b) The initial head CT revealed extensive iso-dense and several hyperdense ill-defined masses. (c) CE-MRI identified diffuse T1 gadolinium-positive (T1 Gd+) lesions. Several high signals on T1 image indicated ITH, and typically hyperintense metastases were identified without significant mass effects. (d) There was negative finding on head CT imaging when the patient was diagnosed with DVT. (e) The last head CT showed diffuse open-ring or hollow circles of hyperintensities with sulci effacement, compatible with diffuse hemorrhagic metastases. The white arrow pointed to a same hemorrhagic metastasis.

Metastatic brain tumor is the hallmark of disseminated end stage disease condition in patients with cancers. At this stage, patients are prone to venous thromboembolism (VTE) and intratumoral hemorrhage (ITH) [1,2,3]. The spontaneous hemorrhagic potential of brain metastases (BMs) contraindicates the routine use of anticoagulants for VTE treatment and prophylaxis. However, increasing evidence suggests no harm of therapeutic anticoagulants and guides the change in recommendation for patients with BMs [2,4,5,6,7,8]. In this study, we report that a patient with innumerable BMs from unknown primary site, previously treated with cabozantinib, developed concurrent multifocal intracerebral hemorrhage after acute treatment of deep vein thrombosis (DVT) by oral rivaroxaban. As far as our concern, the understanding of rivaroxaban-associated ITH is relatively limited in patients with BMs, and the unique clinical presentation of this case may contribute to further understanding of this situation. We provided the timeline for disease progression and reviewed literature for the possible risk factors (Figure 1a).

## 2 Case presentation

A 57-year-old woman was diagnosed with multiple lung, bone, and BMs from unknown primary cancer origin by a cancer center and refused any invasive procedures to confirm tumor pathology. No other medical history had been recorded. As a compromise, the patient agreed to receive a liquid biopsy, which confirmed a CCDC6-RET rearrangement in circulating tumor DNA. An oncologist recommended the regimen of whole brain radiotherapy (WBT) in combination with targeted therapy to control systemic disease progression. However, WBT was denied by the patient herself and family because of the concern of neurological complications in exposing to radiation, even though the probable survival benefit was repeatedly emphasized. Targeted therapy was the only acceptable therapeutic option, and the treatment began with cabozantinib, an oral inhibitor against the tyrosine kinase domain of CCDC6-RET fusion, at a dosage of 60 mg daily. The chief complaints of this patient were frequent fatigue, multiple bone pain, and occasional dizziness. No neurological deficits were found on the initial physical examination. After 17 months of medication, the patient visited our emergency room (ER) for the first time and presented with a 1-month history of gradual neurologic decline involving confusion, memory loss, and imbalance. A head computed tomography (CT) performed without the administration of contrast material revealed extensive iso-dense and several hyperdense ill-defined mass located supratentorially at the junction of gray and white matters. Only sporadic minor peritumoral edema and ITH or calcification were observed at the initial CT scan (Figure 1b). The patient was assigned to an observation room for subsequent contrast-enhanced magnetic resonance imaging (CE-MRI). After 3 days, a gadolinium-based CE-MRI was performed, and during this period, no further deterioration in clinical status was encountered. Contrasted MRI showed innumerable avid enhancements of the parenchymal metastases, which distribute across brain lobes, cerebrum, cerebellum, and brain stem. Sporadic hemorrhagic other than calcified metastases were confirmed on T1, which was indicated by intrinsic high signal (Figure 1c). Only mild peritumoral edema was identified on T2, and the use of mannitol or corticosteroids was suspended. As a concern of disease progression at the primary and metastatic sites, a tissue biopsy and WBT were recommended and again denied. The patient was then discharged in a week. After taking cabozantinib for 26 months, this patient was transferred to our ER in a coma with a Glasgow Coma Scale score of 3 (E1 V1 M1). This patient had a 7-day history of conscious decline and was found to be unresponsive for 2 h. Before 2 weeks, because of a swelling right lower limb for over 2 months, DVT was diagnosed at our department of vascular surgery by compression ultrasonography (CUS). The laboratory test revealed no coagulopathy, thrombocytopenia, or severe liver/renal dysfunction. The D-dimer level was 6,950 ng/mL (normal level: <500). A head CT was scheduled and no hemorrhagic BMs was identified (Figure 1d). Oral rivaroxaban was prescribed at an initial treatment dosing of 15 mg twice a day for 3 weeks. However, after anticoagulation with rivaroxaban for 7 days, this patient confronted with a declined consciousness and subsequent comatose, highly suspected to be the major bleeding complication of intracranial hemorrhage (ICH). Laboratory test revealed the D-dimer level was 3,620 ng/mL. The prothrombin time and international normalized ratio were slightly elevated, and platelet count was normal. The head CT scan confirmed the diagnosis and revealed the extensive but separate foci of intraparenchymal hemorrhage and general brain swelling (Figure 1e). Based on her previous history of extensive BMs, the open-ring or hollow circles of hyperintensities which occupied the majority of hemorrhagic sites were suspected to be hemorrhagic metastases. The patient developed dyspnea right after the CT scan. However, after discussions with the patient’s family, the goals of care were shifted toward comfort measures and no intubation was performed.


**Consent for publication:** Written informed consent for publication of their clinical details and/or clinical images was obtained from the daughter of the patient. A copy of the consent form is available for review by the Editor of this journal.
**Ethics approval and consent to participate:** The CARE guidelines were thoroughly followed to present this case.

## 3 Discussion

BMs are the most common intracranial tumors, and the incidence is estimated at least three times the number of newly diagnosed primary malignant brain tumors [9]. The most frequent primary sites for BMs are lung cancer, breast cancer, and melanoma, accounting for 67–80% of all cancers [10,11]. In patients with four or more BMs, incidence in patients was negatively correlated with the number of metastases, and only less than 5% of patients were diagnosed with 10 or more BMs [12]. With regard to this patient, countless number of BMs is a very rare performance and passive attitude to receive standard treatment could exacerbate the disease progression. CCDC6-RET rearrangement identified in circulating tumor DNA provided an acceptable option of cancer management, and the patient settled for targeted treatment. Cabozantinib inhibits tyrosine kinase activity of RET, MET, VEGFR1, etc. and is approved for the treatment of patients with progressive, metastatic medullary thyroid cancer, advanced renal cell carcinoma with/without history of anti-angiogenic therapy, and hepatocellular carcinoma [13,14]. Phase 3 clinical trials on cabozantinib have been conducted in various malignancies but not in primary or secondary brain tumor. Some sporadic reports showed the potential of cabozantinib in treating BMs. A brief report showed promising intracranial activity of cabozantinib in MET-positive lung cancer with BMs [15]. Another report indicated that cabozantinib was able to reach brain tumors and induce significant regression in two patients with radioresistant BMs from renal cell carcinoma [16]. Therefore, it is likely that this patient could benefit from cabozantinib treatment at the primary and metastatic tumors, though histopathology remains unknown.

Recent advances in chemotherapy and targeted therapies have significantly prolonged the life expectancy in patients with systemic malignancy, but in the meantime, the lifetime risks to develop BMs and cancer-associated comorbidities increase [17]. Patients with intracranial tumors are at greater risk for thrombotic complications and ICH, which accelerates the natural course to the end-of-life period. Significant ICH is reported to occur in 20–50% of patients with BMs [2]. Therefore BMs are considered to be a bleeding risk factor and excluded from most of the selected anticoagulant trials. Another leading cause of morbidity and mortality in this population is the thrombotic complications. Active cancer is a well-established risk factor for DVT and pulmonary embolism, collectively referred to as VTE. Thromboprophylaxis and remedy with anticoagulants may be offered by clinician to selected high-risk cancer patients who will inevitably suffer from a high rate of VTE recurrence and bleeding complications [4]. A meta-analysis focused on ICH in patients with brain tumors receiving therapeutic anticoagulation, and the conclusion is that no significantly increased risk of ICH in patients with BMs [7]. The safety of long-term anticoagulation in 125 patients with BMs has been reviewed by a retrospective study, and the result demonstrated that the incidence of ICH did not increase with the use of anticoagulant therapy [8]. A matched cohort study on ICH in patients with BMs treated with therapeutic enoxaparin concluded that therapeutic anticoagulation in such population did not increase the risk of ICH, but there was four-fold higher about the risk of ICH in patients with melanoma or renal cell carcinoma [2]. The American Society of Clinical Oncology recommends that intracranial malignancy or BMs should not be regarded as an absolute contraindication for therapeutic anticoagulation [6]. Rivaroxaban is a potent NOAC with the high selectivity of direct factor Xa inhibition, and it is recommended in cancer patients for prevention and treatment of VTE [18]. The EINSTEIN program demonstrated that a single drug, rivaroxaban, offered the benefit-to-risk profile of anticoagulation in the short-term and continued treatment of symptomatic venous thrombosis [19]. The CASSINI trial provided information regarding the non-superiority in lowering the incidence of VTE or death because of VTE in high-risk ambulatory patients with cancer. However, during the 180-day trial period, rivaroxaban led to a substantially lower incidence of such events, with a low incidence of major bleeding [20]. As the aforementioned concern of anticoagulant-related intracranial bleeding, both trials exclude patients with BMs and whether these population with VTE could safely benefit from rivaroxaban treatment remains to be further investigated.

In our case, the patient received unexpected clinical benefit from cabozantinib treatment. The patient survived over 2 years until the presence of catastrophic ITH or ICH. Prior minor bleeding of BMs, therapeutic anticoagulation, and cabozantinib treatment were possible causes responsible for the eventual multifocal ICH of this patient. Up to 50% of patients with BMs suffered from spontaneous ICH [2], and the initial head CT/MRI imaging of this patient had revealed several minor intratumoral bleedings, indicating the high-risk hemorrhagic potential (Figure 1b and c). Prior hemorrhagic BMs is a relative contraindication in therapeutic anticoagulation, while the active major bleeding is a situation in which anticoagulation should not be given. Therefore after confirmation of DVT by CUS and significantly elevated D-dimer level, the patient received the second head CT and no trace of hemorrhage diminished the concern of hemorrhagic complication of therapeutic rivaroxaban (Figure 1d). Anticoagulant therapy with NOACs is a simple regimen for treating acute DVT without the need for repeated subcutaneous injection and laboratory monitoring. Increasing evidence supports the safety of NOACs in patients with BMs, without significant increase in the risk of ICH, but the risk of spontaneous ICH can reach as high as 50% in this population [2,7,8]. The exact incidence of rivaroxaban-associated ICH in patients with BMs is still unknown, and further clinical studies are required to ascertain whether the risk of harm associated with hemorrhagic complications exceeds the potential benefit from rivaroxaban. Compared with warfarin-associated ICH, relatively smaller volume of hemorrhage, less chance of hematoma expansion, and more favorable outcomes were found in rivaroxaban-associated ICH [21]. The hemorrhagic features of this patient, small ICH and no expansion of hematoma, shared the similar features of rivaroxaban-associated ICH, but the major difference was the unfavorable outcome. We suspected that the onset of ICH occurred within the first week of rivaroxaban treatment, and no special attention was paid to the neurological decline of the patient until the sudden loss of consciousness, which had been 2 weeks after the possible initial symptomatic ICH. This patient was transferred to our ER at a severe condition and received comfort measures after rapid disease exacerbation. Apart from the two major risk factors of hemorrhage, complication of cabozantinib regimen should not be ignored. The safety of cabozantinib has been evaluated by various clinical trials. However, 3–5% of patients receiving cabozantinib treatment are at risk of hemorrhagic events [13,14]. A phase 1 trial of cabozantinib in the newly diagnosed patients with high-grade gliomas reported that 31% of patients experienced the grade 3/4 adverse event of thrombocytopenia. One of the 26 recruited patients encountered with an adverse event of ICH but do not require surgical intervention [22]. In addition, cabozantinib and rivaroxaban are both substrates of CYP3A4 and P-gp, two important factors in drug metabolism [13,14,23]. It is unclear whether the coexistence of both drugs posts any impact on a single drug exposure, increasing the possibility of hemorrhagic complications. It is believed that the oral rivaroxaban may trigger the diffuse hemorrhagic transformation of BMs in this patient, and, to some extent, diffuse hemorrhage-prone BMs and cabozantinib treatment may also contribute to this malignant transformation.

Emerging evidence supports the use of therapeutic anticoagulants in patients with BMs. Because of the miserable prognosis of anticoagulant-associated ICH, more studies with a greater sample size should be performed to address concerns and guide decisions in clinical practice. Besides, sporadic case reports will also provide novel insights for patient stratification. Clinical characteristics on those patients with severe adverse events will help to identify stratification factors for absolute contraindications for anticoagulation. Therefore, it is recommended that clinicians should still be vigilant to balance the desirable and undesirable effects in this setting.

## Abbreviations list

BMs: brain metastases
CE-MRI: contrast-enhanced magnetic resonance imaging
CT: computed tomography
CUS: compression ultrasonography
DVT: deep vein thrombosis
ER: emergency room
ICH: intracranial hemorrhage
ITH: intratumoral hemorrhage
NOACs: non-vitamin K oral anticoagulants
VTE: venous thromboembolism
WBT: whole brain radiotherapy

## References

[1] Blom JW, Vanderschoot JPM, Oostindiër MJ, Osanto S, Van Der Meer FJM, Rosendaal FR. Incidence of venous thrombosis in a large cohort of 66,329 cancer patients: results of a record linkage study. J Thromb Haemost. 2006;4:529–35. 10.1111/j.1538-7836.2006.01804.x. 16460435

[2] Donato J, Campigotto F, Uhlmann EJ, Coletti E, Neuberg D, Weber GM, et al. Intracranial hemorrhage in patients with brain metastases treated with therapeutic enoxaparin: a matched cohort study. Blood. 2015;126:494–9. 10.1182/blood-2015-02-626788. PMC4548746 25987658

[3] Navi BB, Reichman JS, Berlin D, Reiner AS, Panageas KS, Segal AZ, et al. Intracerebral and subarachnoid hemorrhage in patients with cancer. Neurology. 2010;74:494–501. 10.1212/WNL.0b013e3181cef837. PMC2830918 20142616

[4] Timp JF, Braekkan SK, Versteeg HH, Cannegieter SC. Epidemiology of cancer-associated venous thrombosis. Blood. 2013;122:1712–23. 10.1182/blood-2013-04-460121. 23908465

[5] Di Nisio M, van Es N, Büller HR. Deep vein thrombosis and pulmonary embolism. Lancet. 2016;388:3060–73. 10.1016/S0140-6736(16)30514-1. 27375038

[6] Key NS, Khorana AA, Kuderer NM, Bohlke K, Lee AYY, Arcelus JI, et al. Venous thromboembolism prophylaxis and treatment in patients with cancer: ASCO clinical practice guideline update. J Clin Oncol. 2019;38(5):496–520. 10.1200/jco.19.01461. 31381464

[7] Zwicker JI, Karp Leaf R, Carrier M. A meta-analysis of intracranial hemorrhage in patients with brain tumors receiving therapeutic anticoagulation. J Thromb Haemost. 2016;14:1736–40. 10.1111/jth.13387. 27306689

[8] Horstman H, Gruhl J, Smith L, Ganti AK, Shonka NA. Safety of long-term anticoagulation in patients with brain metastases. Med Oncol. 2018;35:43. 10.1007/s12032-018-1101-z. 29497873

[9] Davis FG, Dolecek TA, McCarthy BJ, Villano JL. Toward determining the lifetime occurrence of metastatic brain tumors estimated from 2007 United States cancer incidence data. Neuro Oncol. 2012;14:1171–7. 10.1093/neuonc/nos152. PMC3424213 22898372

[10] Nayak L, Lee EQ, Wen PY. Epidemiology of brain metastases. Curr Oncol Rep. 2012;14:48–54. 10.1007/s11912-011-0203-y. 22012633

[11] Cagney DN, Martin AM, Catalano PJ, Redig AJ, Lin NU, Lee EQ, et al. Incidence and prognosis of patients with brain metastases at diagnosis of systemic malignancy: a population-based study. Neuro Oncol. 2017;19:1511–21. 10.1093/neuonc/nox077. PMC5737512 28444227

[12] Bhatnagar AK, Flickinger JC, Kondziolka D, Lunsford LD. Stereotactic radiosurgery for four or more intracranial metastases. Int J Radiat Oncol. 2006;64:898–903. 10.1016/j.ijrobp.2005.08.035. 16338097

[13] Exelixis Inc. Cometriq (Cabozantinib) [Labeling-Package Insert]. U.S. Food and Drug Administration Website; 2020. https://www.accessdata.fda.gov/drugsatfda_docs/label/2020/203756s009lbl.pdf

[14] Exelixis Inc. Cabometyx (Cabozantinib) [Efficacy-New Indication]. U.S. Food and Drug Administration Website; 2021. https://www.accessdata.fda.gov/drugsatfda_docs/label/2021/208692s010lbl.pdf

[15] Klempner SJ, Borghei A, Hakimian B, Ali SM, Ou SHI. Intracranial activity of cabozantinib in MET exon 14 – positive NSCLC with brain metastases. J Thorac Oncol. 2017;12:152–6. 10.1016/j.jtho.2016.09.127. 27693535

[16] Négrier S, Moriceau G, Attignon V, Haddad V, Pissaloux D, Guerin N, et al. Activity of cabozantinib in radioresistant brain metastases from renal cell carcinoma: Two case reports. J Med Case Rep. 2018;12:1–6. 10.1186/s13256-018-1875-9. PMC6260776 30474572

[17] Johung KL, Yeh N, Desai NB, Williams TM, Lautenschlaeger T, Arvold ND, et al. Extended survival and prognostic factors for patients with ALK-rearranged non-small-cell lung cancer and brain metastasis. J Clin Oncol. 2016;34:123–9. 10.1200/JCO.2015.62.0138. PMC5070549 26438117

[18] Prins MH, Lensing AWA, Brighton TA, Lyons RM, Rehm J, Trajanovic M, et al. Oral rivaroxaban versus enoxaparin with vitamin K antagonist for the treatment of symptomatic venous thromboembolism in patients with cancer (EINSTEIN-DVT and EINSTEIN-PE): A pooled subgroup analysis of two randomised controlled trials. Lancet Haematol. 2014;1:e37–46. 10.1016/S2352-3026(14)70018-3. 27030066

[19] Bertoletti L, Mismetti P. Oral rivaroxaban for symptomatic venous thromboembolism. N Engl J Med. 2010;363:2499–5. 10. 10.1056/NEJMoa1007903. 10.1056/NEJMoa1007903 21128814

[20] Khorana AA, Soff GA, Kakkar AK, Vadhan-Raj S, Riess H, Wun T, et al. Rivaroxaban for thromboprophylaxis in high-risk ambulatory patients with cancer. N Engl J Med. 2019;380:720–8. 10.1056/NEJMoa1814630. 10.1056/NEJMoa1814630 30786186

[21] Hagii J, Tomita H, Metoki N, Saito S, Shiroto H, Hitomi H, et al. Characteristics of intracerebral hemorrhage during rivaroxaban treatment: comparison with those during warfarin. Stroke. 2014;45:2805–7. 10.1161/STROKEAHA.114.006661. 25082810

[22] Schiff D, Desjardins A, Cloughesy T, Mikkelsen T, Glantz M, Chamberlain MC, et al. Phase 1 dose escalation trial of the safety and pharmacokinetics of cabozantinib concurrent with temozolomide and radiotherapy or temozolomide after radiotherapy in newly diagnosed patients with high-grade gliomas. Cancer. 2016;122:582–7. 10.1002/cncr.29798. 26588662

[23] Janssen Pharms. Xarelto (Rivaroxaban) [Labeling-Package Insert]. U.S. Food and Drug Administration Website; 2020. https://www.accessdata.fda.gov/drugsatfda_docs/label/2020/202439s031,022406s035lbl.pdf

